# Combined approach of perioperative ^18^F-FDG PET/CT imaging and intraoperative ^18^F-FDG handheld gamma probe detection for tumor localization and verification of complete tumor resection in breast cancer

**DOI:** 10.1186/1477-7819-5-143

**Published:** 2007-12-21

**Authors:** Nathan C Hall, Stephen P Povoski, Douglas A Murrey, Michael V Knopp, Edward W Martin

**Affiliations:** 1 Section of PET, Division of Nuclear Medicine, Department of Radiology, The Ohio State University, Columbus, OH, 43210, USA; 2 Division of Surgical Oncology, Department of Surgery, Arthur G. James Cancer Hospital and Richard J. Solove Research Institute and Comprehensive Cancer Center, The Ohio State University, Columbus, OH 43210, USA

## Abstract

**Background:**

^18^F-fluorodeoxyglucose (^18^F-FDG) positron emission tomography/computed tomography (PET/CT) has become an established method for detecting hypermetabolic sites of known and occult disease and is widely used in oncology surgical planning. Intraoperatively, it is often difficult to localize tumors and verify complete resection of tumors that have been previously detected on diagnostic PET/CT at the time of the original evaluation of the cancer patient. Therefore, we propose an innovative approach for intraoperative tumor localization and verification of complete tumor resection utilizing ^18^F-FDG for perioperative PET/CT imaging and intraoperative gamma probe detection.

**Methods:**

Two breast cancer patients were evaluated. ^18^F-FDG was administered and PET/CT was acquired immediately prior to surgery. Intraoperatively, tumors were localized and resected with the assistance of a handheld gamma probe. Resected tumors were scanned with specimen PET/CT prior to pathologic processing. Shortly after the surgical procedure, patients were re-imaged with PET/CT utilizing the same preoperatively administered ^18^F-FDG dose.

**Results:**

One patient had primary carcinoma of breast and a metastatic axillary lymph node. The second patient had a solitary metastatic liver lesion. In both cases, preoperative PET/CT verified these findings and demonstrated no additional suspicious hypermetabolic lesions. Furthermore, intraoperative gamma probe detection, specimen PET/CT, and postoperative PET/CT verified complete resection of the hypermetabolic lesions.

**Conclusion:**

Immediate preoperative and postoperative PET/CT imaging, utilizing the same ^18^F-FDG injection dose, is feasible and image quality is acceptable. Such perioperative PET/CT imaging, along with intraoperative gamma probe detection and specimen PET/CT, can be used to verify complete tumor resection. This innovative approach demonstrates promise for assisting the oncologic surgeon in localizing and verifying resection of ^18^F-FDG positive tumors and may ultimately positively impact upon long-term patient outcomes.

## Background

For many cancers, the risk of recurrence remains deceptively elevated despite presumption of complete resection at the time of initial surgical management. This implies that occult disease may remain undetected at the time of surgery and resultantly may not be resected by the standard surgical approaches. In this regard, diagnostic ^18^F-fluorodeoxyglucose (^18^F-FDG) positron emission tomography/computed tomography (PET/CT) imaging has become an established method for detecting sites of occult disease in oncology and is widely used in medical and surgical planning of cancer patients.

Breast cancer remains the leading cause of newly diagnosed cases of cancer and the second leading cause of cancer deaths among women in the United States [1]. The current standard of care for surgical management of breast cancer is resection of the primary breast tumor and evaluation of the axillary lymph nodes, which includes axillary lymph node dissection in cases of documented nodal involvement [2]. Subsequent appropriately selected adjuvant therapies (including cytotoxic systemic chemotherapy, anti-estrogen therapy, immunotherapy, and radiation therapy) are important adjuncts to the overall management scheme. Despite these best efforts, relative survival rates for women diagnosed with breast cancer are 88% at five years, 80% at 10 years, 71% at 15 years, and 63% at 20 years [3].

^18^F-FDG PET/CT imaging has demonstrated reasonably good success in detecting locally advanced breast cancer, regional lymph node involvement, and distant metastases [4-8]. The current practice for PET/CT imaging is that of preoperative image acquisition, usually taking place at the time of the original evaluation of the cancer patient, for detecting all potential sites of regional and distant metastatic disease. Nevertheless, it is often difficult to intraoperatively localize tumors that have been previously detected on PET/CT imaging done at the time of the original evaluation of the cancer patient. While this current practice of preoperative image acquisition at the time of the original evaluation provides a static roadmap for guiding the surgical approach of the oncologic surgeon, it does not provide vital real-time intraoperative information on tumor localization and immediate verification of complete tumor resection. Therefore, development and refinement of innovative approaches for perioperatively detecting and intraoperatively directing the oncologic surgeon for the intraoperative identification and removal of all sites of disease may ultimately translate into improved long-term patient outcomes.

In the current report, we describe an innovative combined perioperative and intraoperative approach for the localization of tumors and verification of complete tumor resection utilizing single-dose ^18^F-FDG, perioperative PET/CT imaging, and intraoperative handheld gamma probe detection in two very distinctive cases of advanced breast cancer.

## Methods

### Patients

Two patients with potentially resectable metastatic breast cancer that were evaluated by the surgical oncology service at the Arthur G. James Cancer Hospital and Richard J. Solove Research Institution of The Ohio State University were asked to participate in this study. Written informed consent was obtained from each patient after all aspects of the proposed imaging and detection schema were fully discussed with them. One patient had locally advanced breast cancer, involving both her left breast and her left axillary lymph nodes. The other patient had a history of breast cancer with a solitary metastasis in the left lobe of her liver. Both patients underwent standard preoperative testing, including diagnostic CT scans at the time of their original evaluation, for detecting all potential sites of disease.

### ^18^F-FDG PET/CT Imaging and Handheld Gamma Probe Detection Schema

Each patient underwent a preoperative injection of ^18^F-FDG on the same day as surgery. A dose of 14 to 19 mCi ^18^F-FDG was injected intravenously into a peripheral vein of each patient approximately 120 minutes prior to the anticipated time of surgery. At approximately 75 minutes post-injection of ^18^F-FDG, a preoperative clinical PET/CT scan was obtained (Siemens Biograph 16 PET/CT, Knoxville, TN). PET imaging was immediately preceded by transmission CT (for attenuation correction and anatomic correlation purposes) and was obtained from the base of the skull to the mid thighs. Bed positions were scanned for 3 minutes each, moving the patient through the scanner in a craniocaudad direction. Once the preoperative clinical PET/CT scan was acquired, images were reviewed by the oncologic surgeon and the nuclear medicine physician.

Intraoperatively, all potential sites of tumor were localized via standard visualization and palpation. Additionally, tumor was localized with the assistance of a handheld gamma probe (Neoprobe neo2000 unit, Neoprobe Corporation, Dublin, Ohio). The ^18^F-FDG dose from the preoperative clinical PET/CT scan provided radioactivity that was detected intraoperatively by the handheld gamma probe. Gamma counts of blood pool and normal background tissue were recorded along with gamma counts from metabolically active tumor. The handheld gamma probe was used to assist in resection of the tumor and in assessment of surgical margins of resection. Three sigma criteria was used in determining the threshold for positivity of tissue for the gamma probe. The three sigma criteria defines the threshold for positivity of tissue as the average background activity in normal tissue plus three times the square root of the average background activity. This technique has been used with success in intraoperative gamma probe detection of other radioactive tracers [9].

Once resected, the surgical specimen was transported to the radiology department and placed on top of a paraffin block. Digital photographs of the specimen were obtained for visual correlation of specimen placement on the paraffin block. A two bed position ten minute specimen PET/CT scan was then performed on the surgical specimen. Images were processed and reviewed for quality and presence or absence of hypermetabolic foci that were originally noted in the preoperative clinical PET/CT scan. This process allowed for intraoperative verification of complete tumor resection, as well as for potentially marking concerning areas of hypermetabolic activity that might require special attention from the pathologist. The specimen was then transported back to the operating room in order to be sent to and processed by surgical pathology for standard pathologic evaluation.

Postoperatively, each patient was taken to the post-anesthesia care unit (PACU) for standard postoperative recovery. After postoperative standard stabilization and recovery (at approximately 120 minutes after the completion of the surgical procedure), each patient was transported to the radiology department and was re-imaged with a postoperative PET/CT scan. The postoperative PET/CT scan was done utilizing the same preoperative clinical PET/CT^18^F-FDG dose. No additional ^18^F-FDG was administered. The postoperative PET/CT scan was obtained in a similar fashion as that of the preoperative clinical PET/CT scan, except that only the areas of the body with abnormal hypermetabolic foci on the preoperative PET/CT images were targeted for the field of imaging. Bed positions were scanned for 10 minutes each on the postoperative PET/CT scan compared to 3 minutes on the preoperative clinical PET/CT scan. The postoperative PET/CT scan allowed for re-verification of completeness of tumor resection and for assessment for evidence of residual disease.

## Results

### Patient 1

A 72 year-old Caucasian female presented with a palpable left breast mass. Clinical exam revealed a 4 cm palpable mass in the left subareolar region and a 2.5 cm palpable left axillary mass. These findings were confirmed on mammography and ultrasound. An ultrasound-guided 14-gauge core biopsy was performed to both the left breast mass and the left axillary mass. Both core biopsies revealed poorly differentiated invasive ductal carcinoma. CT scan of the chest, abdomen, and pelvis showed a left breast mass, a left axillary mass, generalized atherosclerotic disease, and several subcentimeter hepatic cysts, but no evidence of intrathoracic, intraabdominal, or intrapelvic metastatic disease. Whole body bone scan showed no osseous metastatic lesions.

**Figure 1:**
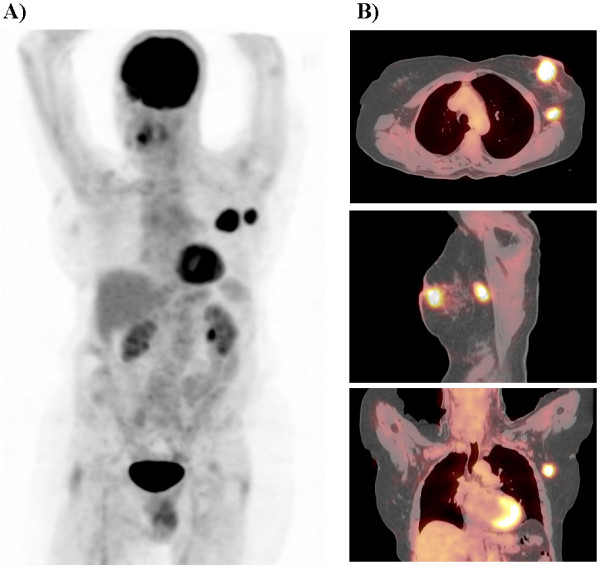
Preoperative PET maximum intensity projection in the lateral projection view (A) and cross sectional fused PET/CT images (B). The preoperative PET/CT scan revealed two hypermetabolic foci, representing the left breast primary tumor and the solitary left axillary metastasis.

On the day of surgery, the preoperative clinical PET/CT demonstrated a solitary hypermetabolic lesion within the left breast with a peak SUV of 14.2 and a solitary hypermetabolic lesion in the left axilla with a peak SUV of 6.6 (Figure 1).

**Figure 2:**
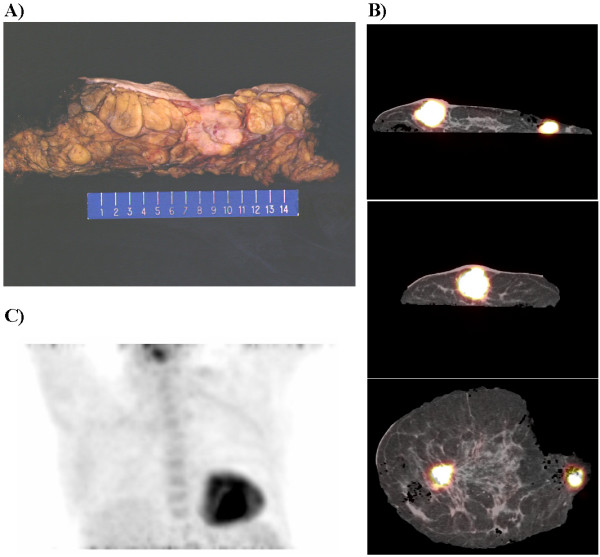
Digital photograph of an axial section of a portion of the specimen of the resected left breast tissue, but devoid of the resected left axillary tissue (A). Cross sectional specimen fused PET/CT images of the resected left breast and left axillary dissection tissue revealing two hypermetabolic foci (B). Postoperative PET maximum intensity projection in the anterior projection view of the chest demonstrating that the primary breast tumor and solitary axillary metastasis have been completely resected and that no residual hypermetabolic foci are demonstrated (C).

The patient was subsequently taken to the operating room. Intraoperative verification of tumor location within the left breast and left axilla was performed by palpation and by handheld gamma probe detection. The patient then underwent a left modified radical mastectomy, including a standard left axillary level I and II lymph node dissection. Post-resection, the surgical bed was again evaluated by handheld gamma probe detection and was found to have no activity above background. The entire resected specimen was re-imaged by specimen PET/CT and revealed two hypermetabolic foci, representing the primary breast tumor and the solitary axillary metastasis (Figure 2A and 2B).

Postoperatively, the patient recovered uneventfully in the PACU. She was subsequently taken to the radiology department and re-imaged with PET/CT scan, demonstrating complete resection of the primary breast tumor and solitary axillary metastasis, and no residual hypermetabolic foci were identified (Figure 2C).

Pathology revealed a 4.5 cm poorly differentiated invasive ductal carcinoma with lymphovascular invasion and negative surgical margins (all greater than 1 cm). Only one of 23 left axillary lymph nodes was involved with metastatic disease (representing the lowest lying lymph node within the left axillary dissection portion of the specimen). This solitary lymph node metastasis measured 2.4 cm in size and there was no evidence of extranodal extension. The primary breast tumor was negative for estrogen receptors (ER) and negative for progesterone receptors (PR). The primary breast tumor was negative for Her2neu by both immunohistochemistry and fluorescence in-situ hybridization (FISH).

### Patient 2

A 52 year-old Caucasian female was originally diagnosed with poorly differentiated invasive ductal carcinoma (pT1c, negative for ER and PR, positive for Her2neu by immunohistochemistry, pN0) of her left breast six years prior to her current presentation. She underwent a left breast lumpectomy, left axillary lymph node dissection, four cycles of postoperative Adriamycin and Cytoxan chemotherapy, and postoperative radiation therapy.

Three years prior to her current presentation, she developed numerous liver metastases and a markedly elevated CA 15-3 at 952 U/mL (normal range 9 to 42). She received six months of Taxotere and Herceptin. Her CA 15-3 decreased into the range of 35 to 40 U/mL and her numerous liver metastases regressed. Due to Taxotere toxicity, she was thereafter maintained on Herceptin alone for the next 18 months. However, 17 months prior to her current presentation, CT scan of the abdomen demonstrated a new left hepatic lesion and brain MRI revealed a solitary 9 × 5 mm enhancing left cerebellar lesion. Gamma knife radiosurgery was successfully performed to the left cerebellar lesion. She was subsequently maintained on Herceptin until 13 months prior to her current presentation when she was noted to have progression of the solitary left hepatic lesion. At that time, she was restarted on combination Taxotere and Herceptin. She did well until more recently when being noted to have a slight rise in CA 15-3 to the 45 to 55 U/mL range. Brain MRI was stable with no lesions. ^18^F-FDG PET/CT scan showed a solitary hypermetabolic left hepatic lesion.

**Figure 3:**
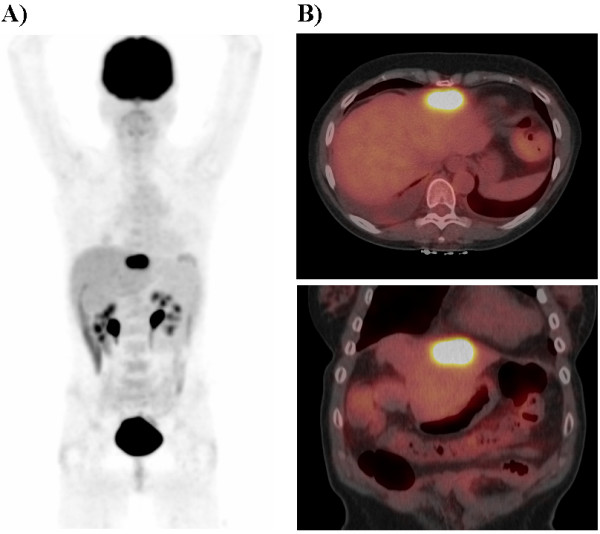
Preoperative PET maximum intensity projection in the anterior projection view (A) and cross sectional fused PET/CT images (B). The preoperative PET/CT scan revealed a solitary hypermetabolic focus in the left lobe of the liver.

**Figure 4:**
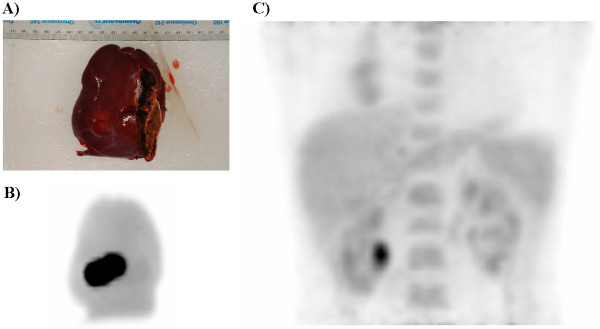
Digital photograph of the resected liver tissue specimen (A) and maximum intensity projection from the specimen PET scan (B). Postoperative PET maximum intensity projection in the anterior projection view of the lower chest and upper abdomen demonstrating that there is no longer a hypermetabolic focus in the region of the liver (C).

On the day of surgery, the preoperative clinical PET/CT demonstrated a solitary hypermetabolic lesion in the left lobe of the liver with a peak SUV of 17.6 (Figure 3). The patient was subsequently taken to the operating room. Intraoperative verification of tumor location within the left hepatic lobe was performed by visualization, palpation, and handheld gamma probe detection. The patient then underwent a left lateral segmentectomy of the liver (Figure 4A). Post-resection, the surgical bed was again evaluated by handheld gamma probe detection and was found to have no activity above background. The entire resected specimen was re-imaged by specimen PET/CT and revealed a single hypermetabolic focus (Figure 4B).

Postoperatively, the patient recovered uneventfully in the PACU. She was subsequently taken to the radiology department and re-imaged with PET/CT scan, demonstrating complete resection of the hypermetabolic hepatic metastasis, and no residual hypermetabolic foci were identified (Figure 4C).

Pathologic evaluation revealed a 3.7 cm carcinoma, with negative surgical margins. The malignant cells were positive for cytokeratin 7 and Her2neu (by FISH) and were negative for cytokeratin 20, ER, PR, BRST-2, and chromogranin.

## Discussion

It is well established that ^18^F-FDG PET imaging is a powerful tool for assisting in the diagnosis as well as in the staging and monitoring of therapy response for a variety of neoplastic processes, including breast cancer [10-12]. One of the main limitations, as it applies to the current use of PET imaging, is that our standard practice of preoperative image acquisition at the time of the original evaluation of the cancer patient cannot realistically be translated into real-time information that is available within the operating room environment. Frequently, it can be difficult for the oncologic surgeon to intraoperatively localize presumed abnormalities that were evident on preoperative image acquisition taken at the time of the initial work-up of the cancer patient. Such limitations can result in the inability to detect occult disease and may lead to incomplete tumor resection at the time of definitive cancer surgery. This may ultimately be responsible for disease recurrence and impact negatively upon long-term patient outcomes. Thus, allowing the oncologic surgeon access to imaging and metabolic information intraoperatively, by using perioperative ^18^F-FDG PET/CT imaging and intraoperative ^18^F-FDG gamma probe detection, has the great potential to significantly improve overall success of complete surgical resection and may ultimately impact positively upon long-term patient outcomes.

Many studies have demonstrated the utility of ^18^F-FDG PET in the staging of breast cancer [4-8,13]. Port et al studied 80 patients with high risk operable breast cancer, comparing conventional imaging staging (bone scan and CT scan) with PET-assisted staging [6]. In this study, PET localized metastatic disease that was not seen with conventional imaging in 5% of patients. Conventional imaging had a higher false positive rate than PET (17% versus 5%), resulting in additional unnecessary tests that were ultimately negative. They concluded that PET was equally sensitive, but more specific, than conventional imaging in detecting occult disease and could better detect metastatic disease, thus resulting in changes in treatment planning in approximately 5% of patients.

Other studies have focused on the utility of ^18^F-FDG PET in assessing axillary lymph node metastases. Kumar et al concluded that ^18^F-FDG PET cannot replace histological staging using sentinel lymph node biopsy in patients with breast cancer, but has both a high specificity and high positive predictive value for the staging of the axilla in these patients [7]. They determined that the positive predictive value and accuracy of ^18^F-FDG PET for the detection of axillary lymph node metastases were 89% and 72%, respectively, for the 80 patients they studied. Unfortunately, due to a false negative rate of 20% in detecting axillary lymph node metastases, it has been concluded that ^18^F-FDG PET cannot replace sentinel node biopsy if the PET scan images are negative within the axilla [4].

It has also been demonstrated that patients with a preoperative ^18^F-FDG PET scan showing axillary lymph nodes that have an SUV greater than 2.3 were more likely to have axillary metastases, thus obviating the need for sentinel lymph node biopsy or needle biopsy to diagnose axillary lymph node involvement [5]. In this setting, Chung et al advocate that the oncologic surgeon can proceed directly to an axillary lymph node dissection to accurately assess the number of axillary lymph nodes involved, eradicate axillary disease, and possibly provide a potential survival benefit [5].

In addition to the usefulness of ^18^F-FDG in the preoperative localization of all sites of tumor, ^18^F-FDG has also been shown to be useful for localizing colorectal tumor intraoperatively using handheld gamma probes [14-19]. Handheld gamma probes have been shown to be capable of detecting the 511 keV photons emitted from positron annihilation [20-22]. Desai et al injected 14 colorectal cancer patients with ^18^F-FDG prior to their surgical procedure [15]. The handheld gamma probe correctly identified a single tumor focus or multiple tumor foci in 13/14 patients (3 standard deviations above counts obtained from normal tissues). This study demonstrated, for the first time, that tumors identified from preoperative whole-body PET scans can be localized during surgery utilizing ^18^F-FDG handheld gamma probe detection. Additionally, Higashi et al have investigated the optimal timing for intraoperative ^18^F-FDG handheld gamma probe detection of tumors using phantom and patient data and determined that detection was best at a duration of time of approximately one to three hours after the injection of ^18^F-FDG [23]. Tumor to normal tissue and tumor to contralateral tissue ratios demonstrated lowest standard deviations at one to three hours compared to later times and there was no significant tumor to normal or tumor to contralateral tissue ratio when probing at later times [23].

Despite the fact that ^18^F-FDG PET is accepted as a useful tool in the preoperative evaluation and staging of appropriately selected breast cancer patients and despite the fact that there is currently some data available on the utilization of the handheld gamma probe for intraoperatively detecting various ^18^F-FDG positive malignancies [14-21,24-27], there has been very little clinical investigation into specifically utilizing a combined approach of perioperative ^18^F-FDG PET/CT technology and intraoperative ^18^F-FDG gamma probe detection. Thus, having available such an innovative combined imaging/detection technology in the perioperative and intraoperative arena has the potential for improving the oncologic surgeons' success rates in achieving an optimal and/or complete resection of the tumor burden. The current report, for the first time, comprehensively describes the feasibility and usefulness of such an innovative combined strategy of perioperative ^18^F-FDG PET/CT imaging and intraoperative ^18^F-FDG gamma probe detection for tumor localization and verification of resection in two distinctive cases of potentially resectable metastatic breast cancer. Such an innovative approach clearly provides the oncologic surgeon with real-time access to metabolic and morphologic tumor information that may better lead to complete tumor resection and avoidance of missed occult disease.

In the two patients that were studied in this paper, ^18^F-FDG PET/CT accurately localized all tumor sites. Acquiring the PET/CT scan immediately prior to the surgical procedure allowed for visual verification of tumor location prior to surgery. The ^18^F-FDG dose used for the preoperative clinical PET/CT scan was adequate for the intraoperative localization of all hypermetabolic tumor foci by the handheld gamma probe. The handheld gamma probe results from the tumor and surrounding tissue correlated well with the PET scan findings. The intraoperative specimen PET/CT and postoperative PET/CT of the patients were highly useful for verifying complete tumor resection.

With the advent of consideration of utilizing ^18^F-FDG for intraoperative gamma probe detection in oncologic surgery, it is not unexpected that increasing questions and concerns will arise with regards to resultant occupational radiation exposure to the operating surgeon and to the involved perioperative personnel. To date, essentially no published data is available that has specifically addressed this particular issue. However, the current authors have very recently published data on calculated theoretical radiation doses encountered by the surgeon during gamma probe surgery [16]. A theoretical maximum estimated radiation dose of only 606 μGy (0.606 mGy) would be incurred by a surgeon standing 0.152 meters (6 inches) from the ^18^F-FDG source for 5 hours. Such a radiation dose is only a fraction of the annual occupational limit of 50,000 μGy/year (50 mGy/year), thus potentially allowing the surgeon to perform approximately 80 such cases a year. Furthermore, a more comprehensive evaluation of actual occupational radiation exposure to the operating surgeon and to the involved perioperative personnel is currently being undertaken by the present authors.

One of the limitations of the innovative techniques presented in this communication is the lack of specificity of ^18^F-FDG. It is well established that normal physiologic uptake of ^18^F-FDG is demonstrated to varying degrees in multiple normal tissues. It is also widely accepted that ^18^F-FDG is excreted in the urine and therefore significant activity is noted within the collecting systems of the kidneys as well as the bladder. These are recognized and understood limitations of ^18^F-FDG PET imaging. However, utilizing the 3 sigma threshold for a gamma probe with normal background activity coming from normal surrounding tissues, as well as consideration being given to location of abnormal activity from the preoperative PET/CT scan, some of these limitations can be overcome. False positive findings on the PET/CT scan and consequently false positive gamma probe investigation findings should certainly be correlated with frozen section and pathology verification. This study was designed to demonstrate the feasibility of an innovative technique involving perioperative and intraoperative imaging and intraoperative gamma probe utilization for assistance in tumor localization and verification of resection.

Another limitation of ^18^F-FDG PET/CT scanning is the 110 minute half-life of ^18^F. This limitation is relavant to postoperative PET/CT scanning, specimen PET/CT scanning, and intraoperative gamma probe detection. By increasing the scan time (10 minutes per bed position), we have been able to generate high quality images on the PET/CT scanner at times as far out as 9 to 10 hours post-injection of the clinical dose of ^18^F-FDG.

The limitations of PET scanning as well as the limitations associated with the specificity of ^18^F-FDG as a tumor tracer are acknowledged. Further development of more specific radiotracers may compensate for many of these limitations. However, addition of these new and innovative technologies to the operative arena have significant potential for improving successful resection of tumors with negative margins and improving long term patient outcomes. Further explorations on the feasibility of these techniques as well as validation with pathology correlation and patient outcomes will be helpful in assessing the benefit of these techniques.

## Conclusion

This innovative combined technology of perioperative ^18^F-FDG PET/CT imaging and intraoperative ^18^F-FDG gamma probe detection demonstrates great promise for providing real-time feedback to the oncologic surgeon for successful complete surgical resection of all sites of tumor. The future potential for a more widespread application of this innovative technology to the surgical oncology arena lies in such applications as verification of primary tumor site resection, margin assessment, regional lymph node assessment, and recognition of sites of occult disease. All these potential applications have great promise for positively impacting on long-term patient outcomes.

## Abbreviations

^18^F-FDG, ^18^F-fluorodeoxyglucose; PET/CT, positron emission tomography/computed tomography; PACU, post-anesthesia care unit; SUV, standardized uptake value; MRI, magnetic resonance imaging

## Competing interests

The author(s) declare that they have no competing interests.

## Authors' contributions

NCH and SPP organized, wrote, and revised the manuscript. EWM was the supervising senior physician for the entire project. IS, DAM, MVK, and EWM assisted in the writing and editing of this manuscript. All of the authors have approved the final version of this manuscript.
